# Tissue distribution and net maintenance and growth requirements of key trace minerals in fattening yaks (*Bos grunniens*) on the Qinghai–Tibetan plateau

**DOI:** 10.3389/fvets.2026.1864547

**Published:** 2026-07-03

**Authors:** Shengchun Xu, Shuting Bao, Jiyuan Zhang, Zhilong Wang, Shujie Liu, Shuxiang Wang, Shatuo Chai, Xun Wang, Yingkui Yang, Jiaying Lv

**Affiliations:** 1 Qinghai University, Xining, Qinghai, China; 2 Key Laboratory of Plateau Grazing Animal Nutrition and Feed Science of Qinghai Province, Xining, Qinghai, China; 3 Yak Engineering Technology Research Center of Qinghai Province, Xining, Qinghai, China; 4 National Yak Technology Innovation Center (Preparatory), Xining, Qinghai, China

**Keywords:** net growth requirements, net maintenance requirements, tissue distribution, trace minerals, yaks (*Bos grunniens*)

## Abstract

**Introduction:**

This study aimed to characterize the tissue distribution of key trace elements in fattening yaks and to establish empty body weight (EBW)-based prediction models for the net growth requirement (NRG) of copper (Cu), manganese (Mn), zinc (Zn), iron (Fe), selenium (Se), and cobalt (Co), as well as to derive their net maintenance requirement (NMR).

**Methods:**

A comparative slaughter plus graded feeding design was used. Forty approximately 4-year-old male yaks (234.20 ± 9.86 kg) were assigned to baseline slaughter (BL), midterm slaughter (M), and terminal slaughter groups stratified by intake: ad libitum (AL), 70% of ad libitum (IR70, n = 8), and 40% of ad libitum (IR40). Tissue concentrations of target elements were determined by ICP-OES and ICP-MS. Body weight and intake data were used to develop EBW-based NRG models and derive NMR.

**Results:**

Cu and Fe were primarily distributed in viscera, Mn predominantly in bone, and Zn, Se, and Co mainly in muscle. For the 230 to 320 kg body-weight range, the EBW-based NRG prediction models were: NRG_Cu_ = 2.7194 × EBW^0.1827^; NRG_Mn_ = 0.1302 × EBW^0.3880^; NRG_Zn_ = 17.1124 × EBW^0.1092^; NRG_Fe_ = 7.1755 × EBW^0.5054^; NRG_Se_ = 0.0034 × EBW^0.8043^; and NRG_Co_ = 0.0077 × EBW^0.0974^. The corresponding NRG values (mg/kg EBW) were Cu 7.08–7.58, Mn 0.99–1.15, Zn 30.31–31.57, Fe 101.21–122.08, Se 0.23–0.31, and Co 0.01. The NMR values (mg/d) were Cu 0.87, Mn 0.45, Zn 14.48, Fe 19.89, Se 0.11, and Co 0.01.

**Discussion:**

EBW-based models provide robust NRG and NMR parameters for Cu, Mn, Zn, Fe, Se, and Co in late-fattening yaks. These yak-specific values offer practical targets for precise mineral supplementation and ration formulation in yak production systems across Asia, helping prevent deficiency or excess and improve production efficiency.

## 1 Introduction

Yak (*Bos grunniens*) is a domesticated species unique to the Qinghai–Tibetan Plateau and adjacent high-altitude regions, renowned as the “ship of the plateau.” It serves both as an economic mainstay for local herders and as an important maintainer of alpine ecosystem function (1). According to China’s National Bureau of Statistics (2019), the yak population on the Qinghai–Tibetan Plateau exceeds 16 million head. Its meat and milk are rich in conjugated linoleic acid (CLA) and omega-3 (ω-3) fatty acids, conferring distinctive nutritional and functional value (2). As yak production shifts from traditional grazing to semi-confinement and even full confinement, the establishment of scientific and standardized feeding systems has become urgent. Given that mineral nutrition is a core determinant of animal health and productivity, its precise provision is a key step toward achieving this goal.

Trace minerals play indispensable roles in antioxidant defense, immune modulation, and rumen function in yaks. Under high-altitude conditions, the biological functions of selenium are particularly prominent; selenium deficiency can markedly reduce serum glutathione peroxidase (GSH-Px) activity, with the magnitude of decline exceeding that in ordinary Holstein cattle by more than twofold (3, 4). Cobalt is an essential element for rumen microorganisms to synthesize vitamin B_12_ (5). Cobalt deficiency blocks the conversion of succinate to propionate, thereby disrupting propionate metabolism (6). However, the natural contents of selenium, cobalt, and related elements in soils and forages on the Qinghai–Tibetan Plateau are relatively low (7), frequently leading to nutritional deficiencies in yaks, such as growth retardation, reduced reproductive performance, and diminished product quality. In practice, feeding remains largely empirical, which readily results in mineral oversupply or undersupply and consequently causes resource waste and metabolic risks.

Although research on mineral nutrition in ruminants is relatively mature internationally, the unique high-altitude adaptation and metabolic characteristics of yaks make it difficult to directly apply standards developed for conventional beef cattle. Existing studies have focused largely on calves or growing animals and mainly on macroelements such as calcium (Ca) and phosphorus (P) (8) or on a limited set of trace elements such as selenium (Se) (9). Systematic evidence for adults or animals in the fattening stage remains insufficient. At present, the requirements for key trace elements such as iron (Fe) and zinc (Zn) are often inferred from mathematical models developed for yak calves (10), leaving uncertainty when extrapolating across body weight ranges and production goals.

In this context, the present study integrates comparative slaughter and graded feeding trials to systematically quantify requirement parameters for trace minerals including Fe, Zn, copper (Cu), manganese (Mn), Se, and cobalt (Co) in adult yaks. The goal is to fill gaps in current standards and provide actionable quantitative evidence to support precise mineral supplementation and science-based feeding of yaks on the Qinghai–Tibetan Plateau.

## 2 Materials and methods

### 2.1 Sample size selection

This experiment used the comparative slaughter method of Lofgreen and Garrett (11). Considering operational complexity, cost, and animal ethics (3R principles), 8 animals were allocated per group, from which 5 representative individuals were selected for slaughter to ensure within-group homogeneity (12). All animals were screened at trial start, with coefficients of variation for breed, age, and initial body weight controlled within 5% to minimize inter-individual variation.

### 2.2 Experimental design

The trial was conducted from September to December 2023 in Haiyan County, Haibei Tibetan Autonomous Prefecture, Qinghai Province, China. After a 15-day pretrial period for adaptation to housing conditions and experimental diets, determination of voluntary feed intake, and exclusion of abnormal individuals, forty healthy 4-year-old male Huanhu yaks at the late fattening stage with similar body weight (234.20 ± 9.86 kg) were allocated to three groups using a randomized block design: a baseline slaughter group (baseline slaughter, BL; 8 head), a midterm slaughter group (M, *n* = 8), and a terminal slaughter group (*n* = 24). The terminal group was further divided into an ad libitum group (AL, *n* = 8), a 70% intake-restricted group (IR70, *n* = 8), and a 40% intake-restricted group (IR40, *n* = 8).

Slaughtering was scheduled as follows: at day 0, five yaks from the BL group were slaughtered; at day 45, five yaks from the M group were slaughtered; and at day 90, fifteen yaks from the terminal cohort were slaughtered, comprising five animals per AL, IR70, and IR40 group. Throughout the trial, all yaks had free access to water. The M and AL groups were fed ad libitum, with approximately 10% of feed remaining in the trough to ensure ad libitum intake. The IR70 and IR40 groups received 70 and 40%, respectively, of the previous day’s average feed intake of the AL group, with daily ration adjustments to ensure that actual intake closely matched the target levels.

The BL, M, and AL groups served as time-course controls representing the initial, midterm, and ad libitum terminal slaughter points, respectively, while the AL, IR70, and IR40 groups at the terminal slaughter formed a graded intake comparison corresponding to 100 percent, 70 percent, and 40 percent of dry matter intake (DMI, kg/d).

### 2.3 Feeding management and diets

All yaks were intact males and received the necessary vaccinations before the start of the trial. Each animal was assigned a unique identification number. Animals in each group were housed in individual pens and fed individually. All yaks were offered the same basal diet with free access to water and were fed a total mixed ration (TMR) twice daily at 08:00 and 17:00. The diet was formulated according to China’s *Breeding Standard for Beef Cattle* (13). The nutrient composition and levels of the experimental diet are shown in Table 1, and the trace mineral concentrations are shown in Table 2.

**Table 1:** Basic diet composition and nutritional level (DM basis).

Feeds composition		Nutritional level	
Ingredients	Ratio (%)	Items	Content
Oats hay	35.00	Dry matter (%)	91.72
Corn	29.75	Metabolizable energy^b^ (MJ/kg)	11.89
Wheat	8.39	Crude protein (%)	13.18
Wheat bran	8.56	Ether extract (%)	4.97
Rapeseed dregs	8.55	Neutral detergent fiber (%)	31.36
Soybean meal	2.91	Acid detergent fiber (%)	18.36
Palm oil fat powder	2.84	Ca (%)	0.56
CaHPO_4_	1.00	P (%)	0.48
NaCl	1.00		
Premix^a^	2.00		
Total	100.00		

a Premixed ingredients provided VA 4000 IU, VD 500 IU, VE 40 IU, Mn 30 mg, Fe 65 mg, Co 0.1 mg, Cu 10 mg, Zn 25 mg, Se 0.1 mg and I 0.5 mg per kg of ration.

b Metabolic energy is calculated (35), and the rest are measured values.

**Table 2:** Major trace mineral content in the diet (DM basis).

Trace elements	Content (mg/kg)
Cu	15.51
Mn	71.63
Zn	59.61
Fe	143.10
Se	0.15
Co	0.16

### 2.4 Sample collection and slaughter procedures

#### 2.4.1 Sample collection from feeds and body weight records

At the beginning and end of the experiment, 200 g samples were taken from the experimental diet using the quadrant method (14). The samples were then ground using a grinder to a particle diameter of 1 mm for routine feed analysis. The crude protein content in the feed was measured using the Kjeldahl method (Kjeltec™ 8,400, Foss Analytical A/S, Denmark), and crude fat was determined by Soxhlet extraction, both according to AOAC official methods (15). Calcium and phosphorus levels were determined using inductively coupled plasma optical emission spectrometry (ICP-OES, Thermo Fisher Scientific, USA). The concentrations of neutral detergent fibre (NDF) and acid detergent fibre (ADF) were assessed according to the Van Soest et al. (16) detergent fibre analysis method. At the same times, yaks were weighed, and daily feed intake and orts were recorded.

#### 2.4.2 Slaughter procedures

At the end of the trial, slaughter was performed following the procedures of Galvani et al. (17). Feed was withdrawn for 16 h and water for 2 h starting at 16:00 on the afternoon before slaughter. Immediately before stunning, pre-slaughter live weight was recorded as shrunk body weight (SBW, kg). Animals were electrically stunned and exsanguinated via the jugular vein, and whole blood was collected. Skinning was performed along the ventral midline, followed by removal of the head, feet, tail, heart, lungs, kidneys, reproductive viscera, and the gastrointestinal tract. Gastrointestinal contents were emptied, the tract was rinsed and drained, and the tract was weighed again; the difference between the two weights was taken as digesta weight. Empty body weight (EBW, kg) was calculated as SBW minus digesta weight.

Carcasses were split along the midline. Bone, muscle, and fat were separated, and all fat was collected and weighed, including mesenteric fat, intestinal fat, carcass fat, and perivisceral fat. The head was sawn along the midline and weighed; the hide was removed from the head, and soft tissues were separated from bone. The body hide was first roughly trimmed manually and then refined with an electric clipper; hair and hide were weighed separately.

#### 2.4.3 Sample preparation and storage

Sample pooling for chemical analysis was conducted as follows: Bone samples consisted of the skeleton from the right half of the carcass, the right side of the skull, the bones of the right half of the tail, and the distal bones of the right fore- and hindlimbs. Muscle samples were pooled from the right half of the carcass, the right head musculature, the musculature of the right half of the tail, and the right fore- and hindlimbs. Adipose samples were prepared by proportionally mixing fat from the right carcass, right head, right half of the tail, and right fore- and hindlimbs, together with 50% of the gastrointestinal fat (mesenteric and intestinal fat) and 50% of the visceral fat (pericardial, perirenal, and perihepatic fat).

Visceral samples (including the heart, liver, lungs, spleen, kidneys, and emptied gastrointestinal tissues) were ground and combined with the collected blood. The hide and hair were separated using electric clippers, and hide samples were frozen prior to grinding to facilitate fragmentation. After grinding and homogenization, approximately 500 g of each tissue type (bone, muscle, adipose, viscera–blood mixture, hide, and hair) was subsampled in triplicate and stored at −20 °C for subsequent analysis.

### 2.5 Measured variables and analytical methods

Feed samples were dried in a forced-air oven at 105 °C until constant weight to determine dry matter (DM) content (18). Dry matter of animal tissues was determined by vacuum freeze-drying (LGJ-12, Beijing Songyuan Huaxing Technology Development Co., Ltd., China). The lyophilized samples were then ground and homogenized to prepare uniform test portions. After microwave-assisted digestion, mineral elements in the samples—including copper (Cu), manganese (Mn), zinc (Zn), iron (Fe), selenium (Se), cobalt (Co), and chromium (Cr)—were determined using inductively coupled plasma optical emission spectrometry (ICP-OES, Thermo Fisher Scientific, USA) and inductively coupled plasma mass spectrometry (ICP-MS, Thermo Fisher Scientific, USA). Final reported concentrations were assigned as follows: for elements with measured concentrations greater than 100 μg/L, ICP-OES results were used; for elements with measured concentrations less than 100 μg/L, ICP-MS results were used. The main operating parameters for ICP-OES and ICP-MS are provided in Supplementary Tables S1, S2, respectively. Quantification of each element on the corresponding instrument was based on its calibration curve; linear regression equations and correlation coefficients are listed in Supplementary Table S3.

### 2.6 Prediction models for net growth requirements (NRG)

According to the Agricultural Research Council (ARC) (19), whole-body mineral content can be derived using a log-allometric growth model that relates EBW (kg) of the comparative slaughter groups to whole-body mineral content of the trace element y (mg), as shown in Equation 1:


$$\log_{10}y=a+b\times \log_{10}\mathrm{EBW}$$ (1)

Where: *y* is the whole-body mineral content after removal of gastrointestinal contents (mg); *a* is the intercept; *b* is the regression coefficient.

Equation 2 was obtained by algebraic transformation and differentiation of Equation 1 and was used to estimate the mineral requirement per unit increase in EBW at different EBW values:


$$y^{\prime }=b\times 10^{a}\times {\mathrm{EBW}}^{b-1}$$ (2)

Where: *y*′ is the mineral element required per unit increase in EBW (mg/kg EBW); *a* and *b* are as defined in Equation 1.

To infer the mineral required per unit of body weight gain, the ratio of body weight (BW, kg) to EBW (kg) is used for conversion, yielding Equation 3:


EBW=d+c×BW (3)

Where: *d* is the intercept; *c* is the regression coefficient.

### 2.7 Prediction models for net maintenance requirements (NMR)

Using Equations 1 and 3, initial whole-body mineral content was predicted from initial body weight for all groups. Mineral deposition was calculated as the difference between final and initial content. Maintenance requirements were estimated using the factorial approach (19), which partitions utilization between maintenance and deposition. Linear regression was established between daily mineral deposition (mg/d) and DMI (kg/d). When DMI is zero, mineral intake ceases but endogenous losses persist via intestinal secretions, urine, and dermal routes. The negative intercept at zero DMI quantifies these obligatory losses; its absolute value represents the net maintenance requirement, defined as the mineral quantity that must be absorbed to offset these losses and maintain balance.

### 2.8 Data statistical analysis

Data were compiled in Excel 2021 (Microsoft Corp., Redmond, WA, USA) and analyzed with SPSS 27.0 (IBM Corp., Armonk, NY, USA). Normality was examined using the Shapiro–Wilk test and homogeneity of variances with Levene’s test; any variable violating assumptions (*p* < 0.05) was log-transformed prior to analysis. One-way ANOVA followed by Duncan’s multiple range test was employed with significance declared at *p* < 0.05. Results are presented as means ± SEM.

The construction of the linear regression model was implemented using R language and its relevant packages. Model goodness-of-fit was evaluated through the coefficient of determination (*R*^2^), root mean square error (RMSE), and statistical significance of regression coefficients. Furthermore, leave-one-out cross-validation (LOOCV) was employed to validate model robustness. Predictive performance of the model was comprehensively assessed using cross-validation root mean square error (RMSECV) and cross-validation coefficient of determination (*R*^2^CV).

## 3 Results

### 3.1 Effects of slaughter stage and feeding level on body weight and growth performance in fattening yaks

As shown in Table 3, IBW did not differ significantly among groups (*p* > 0.05). DMI differed significantly among groups, following the order AL > M > IR70 > IR40 > BL (*p* < 0.05). Across slaughter stages, fattening yaks showed the same pattern of change for final body weight (FBW), SBW and EBW, with the order being AL > M > BL (*p* < 0.05).

**Table 3:** Effects of slaughter stage and feeding level on body weight and growth performance in fattening yaks.

Items	Groups^a^					SEM	*p*-value
	BL	M	AL	IR70	IR40		
Initial body weight (IBW, kg)	238.56	227.62	237.58	235.52	228.78	1.97	0.455
Final body weight (FBW, kg)	240.20^d^	264.80^c^	320.20^a^	310.40^a^	288.80^b^	6.36	<0.001
Average daily gain (ADG, g/d)	—	826.67^a^	918.00^a^	832.00^a^	666.89^b^	64.29	<0.001
Dry matter intake (DMI, kg/d)	0^e^	7.10^b^	7.83^a^	5.89^c^	3.47^d^	0.58	<0.001
Shrunk body weight (SBW, kg)	227.60^d^	257.20^c^	306.80^a^	296.80^a^	276.40^b^	6.12	<0.001
Empty body weight (EBW, kg)	192.16^d^	226.33^c^	272.39^a^	261.62^a^	241.86^b^	6.10	<0.001

a Different lowercase letters above the same row indicate significant differences (*p* < 0.05), whereas identical letters or no letter indicate no significant difference (*p* > 0.05).

BL, baseline slaughter group; M, midterm slaughter group; AL, ad libitum terminal group; IR70, 70% of ad libitum intake group; IR40, 40% of ad libitum intake group.

Under different feeding levels, FBW, ADG, SBW, and EBW in the AL and IR70 groups were significantly higher than those in the IR40 group (*p* < 0.05), whereas no significant difference was observed between the AL and IR70 groups (*p* > 0.05).

### 3.2 Tissue distribution of key trace minerals in fattening yaks

**Figure 1:**
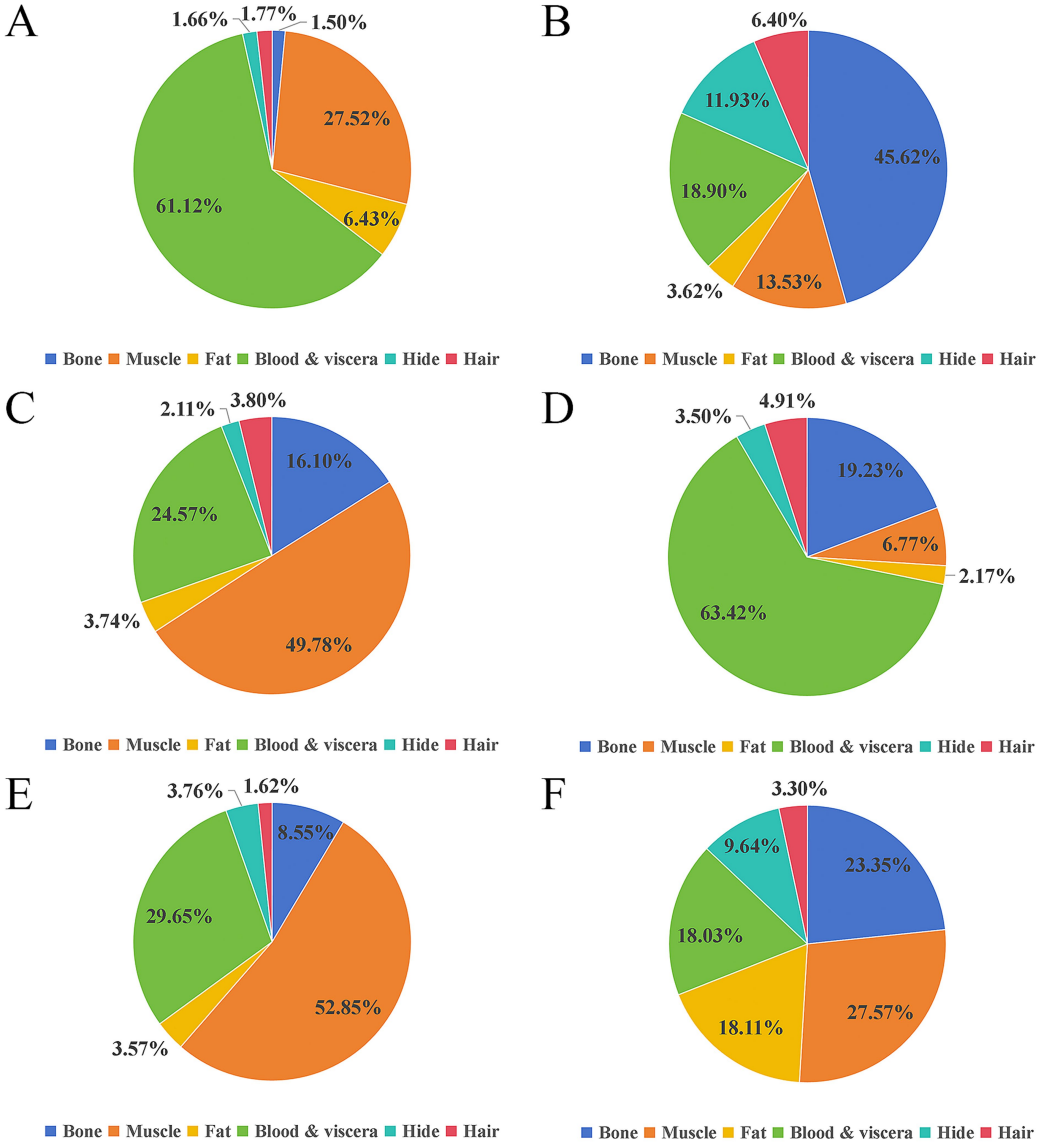
Distribution of trace elements in fattening yak tissues. **(A)** Copper (Cu), **(B)** manganese (Mn), **(C)** zinc (Zn), **(D)** iron (Fe), **(E)** selenium (Se), **(F)** cobalt (Co).

Based on tissue weights (Supplementary Table S4), tissue dry matter content (Supplementary Table S5), and mineral concentrations, the tissue distribution of trace minerals in fattening yaks was calculated. Copper was enriched in viscera (including blood) and muscle, accounting for 61.12 and 27.52% of the whole-body total, respectively (Figure 1A; Supplementary Table S6). Manganese was mainly in bone and viscera (including blood), at 45.62 and 18.90% (Figure 1B; Supplementary Table S7). Zinc was highest in muscle and viscera (including blood), at 49.78 and 24.57% (Figure 1C; Supplementary Table S8). Iron was primarily stored in viscera (including blood) and bone, at 63.42 and 19.23% (Figure 1D; Supplementary Table S9). Selenium was largely in muscle and viscera (including blood), at 52.85 and 29.65% (Figure 1E; Supplementary Table S10). Cobalt was mainly in muscle and bone, at 27.57 and 23.35% (Figure 1F; Supplementary Table S11). Across different slaughter stages and feeding-level groups, the proportions of trace minerals in various tissues showed some numerical variation, but these differences were not statistically significant overall (*p* > 0.05).

### 3.3 Net growth requirements and prediction models for major trace minerals in fattening yaks

**Figure 2:**
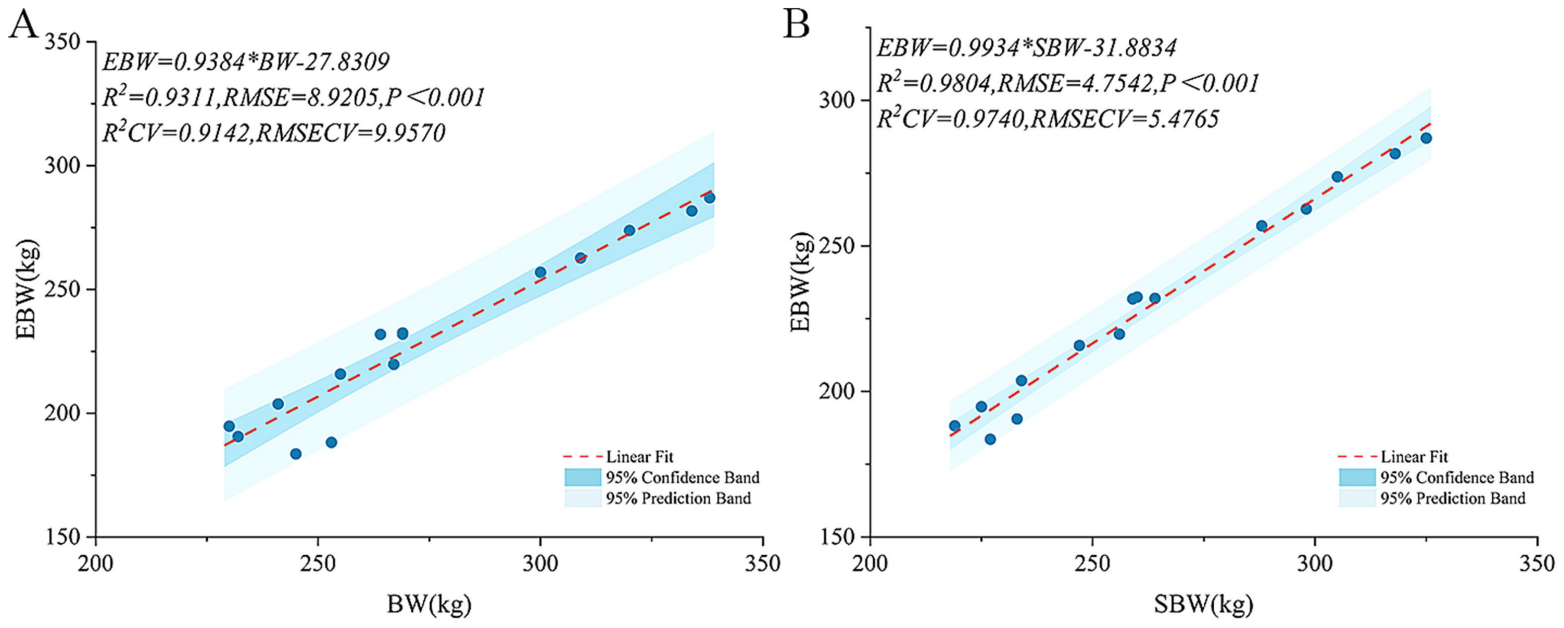
Linear regression relationships of empty body weight (EBW) with body weight (BW) and shrunk body weight (SBW) in yaks. **(A)** EBW versus BW; **(B)** EBW versus SBW.

**Figure 3:**
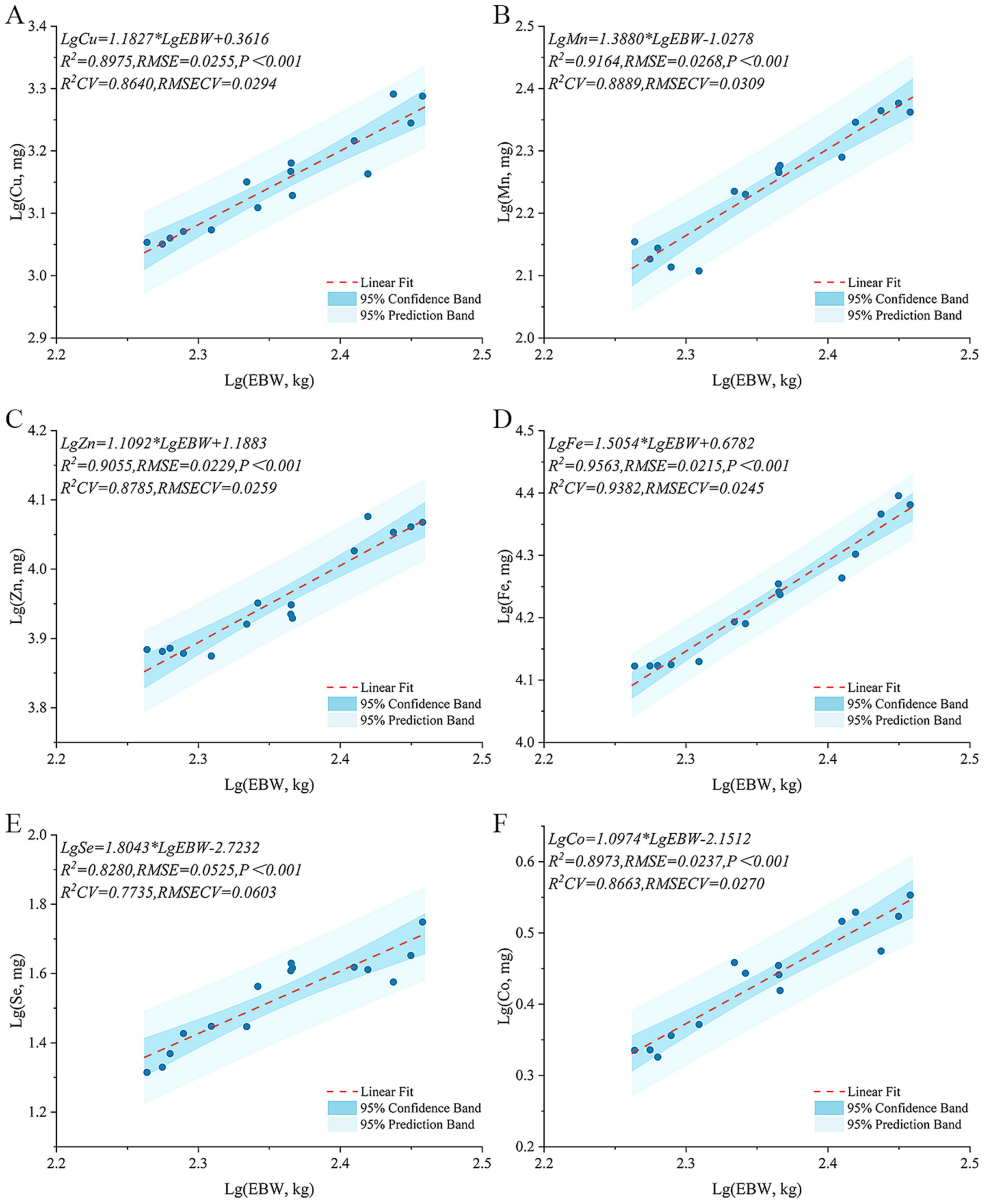
Log10-linear regression relationships between empty body weight (EBW) and total body content of trace minerals in yaks during the fattening period: **(A)** Copper (Cu), **(B)** manganese (Mn), **(C)** zinc (Zn), **(D)** iron (Fe), **(E)** selenium (Se), **(F)** cobalt (Co).

As shown in Figure 2, EBW was strongly linearly correlated with both BW (*R*^2^ = 0.9311, *R*^2^CV = 0.9142, RMSECV = 9.9570) and SBW (*R*^2^ = 0.9804, *R*^2^CV = 0.9740, RMSECV = 5.4765), indicating that EBW can be effectively predicted from either BW or SBW. In addition, the logarithm of EBW was highly correlated with the logarithm of whole-body mineral content (*R*^2^ = 0.8280–0.9563, *R*^2^CV = 0.7735–0.9382, RMSECV = 0.0245–0.0603; Figure 3), supporting the use of EBW to predict whole-body mineral content in yaks. These cross-validation results further indicate that the prediction models had acceptable robustness and predictive reliability.

By differentiating the equations in Figure 3, prediction equations for net growth requirements were obtained (Table 4). For fattening yaks weighing 230–320 kg, the growth requirements were: Cu 7.08–7.58 mg/kg EBW, Mn 0.99–1.15 mg/kg EBW, Zn 30.31–31.57 mg/kg EBW, Fe 101.21–122.08 mg/kg EBW, Se 0.23–0.31 mg/kg EBW and Co 0.01 mg/kg EBW.

**Table 4:** Net growth requirements (NRG) and prediction models for minerals in fattening yaks.

Items	BW (kg)				Equations^a^
	230	260	290	320	
EBW (kg)	188.00	216.53	244.31	272.46	—
Cu (mg/kg EBW)	7.08	7.26	7.43	7.58	NRG_Cu_ = 2.7194 × EBW^0.1827^
Mn (mg/kg EBW)	0.99	1.05	1.10	1.15	NRG_Mn_ = 0.1302 × EBW^0.3880^
Zn (mg/kg EBW)	30.31	30.79	31.19	31.57	NRG_Zn_ = 17.1124 × EBW^0.1092^
Fe (mg/kg EBW)	101.21	108.70	115.54	122.08	NRG_Fe_ = 7.1755 × EBW^0.5054^
Se (mg/kg EBW)	0.23	0.26	0.28	0.31	NRG_Se_ = 0.0034 × EBW^0.8043^
Co (mg/kg EBW)	0.01	0.01	0.01	0.01	NRG_Co_ = 0.0077 × EBW^0.0974^

a BW, body weight; EBW, empty body weight; NRG, net growth requirement.

### 3.4 Daily mineral deposition in yaks and prediction of net maintenance requirements

**Figure 4:**
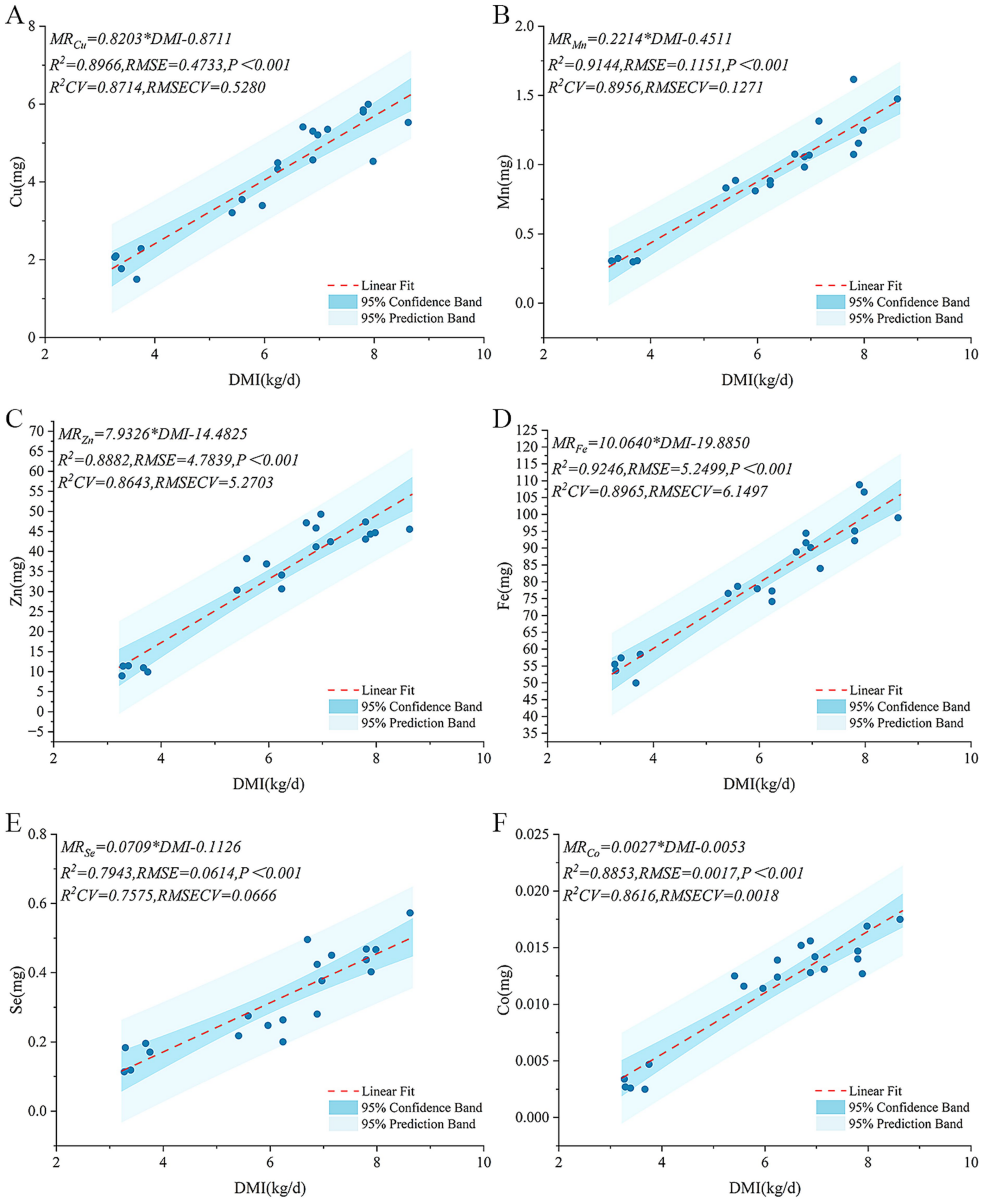
Linear regression relationships between daily mineral retention (MR) and dry matter intake (DMI) in fattening yaks: **(A)** Copper (Cu), **(B)** manganese (Mn), **(C)** zinc (Zn), **(D)** iron (Fe), **(E)** selenium (Se), **(F)** cobalt (Co).

Based on the conversion equation between EBW and BW established in Figure 2 and the logarithmic relationship between EBW and whole-body contents of major trace elements shown in Figure 3, whole-body mineral contents at the beginning of the experiment were predicted for groups M, AL, IR70, and IR40. Daily mineral retention (MR; Supplementary Table S12) during the experiment was calculated as the difference in whole-body mineral content between the end and the start of the trial divided by the corresponding number of days. Linear regression was performed between MR and DMI, and the negative intercept of the regression equation was defined as NMR (net maintenance requirement). Figure 4 presents the coefficients of determination of the prediction equations (*R*^2^ = 0.7943–0.9246, *R*^2^CV = 0.7575–0.8965, RMSECV = 0.0018–6.1497), indicating strong correlations and supporting the feasibility of predicting mineral NMR using DMI and daily MR. These cross-validation results further suggest that the prediction models had acceptable robustness and predictive performance. Table 5 also presents the predicted NMR for the trace minerals: Cu 0.87 mg/d, Mn 0.45 mg/d, Zn 14.48 mg/d, Fe 19.89 mg/d, Se 0.11 mg/d, and Co 0.01 mg/d.

**Table 5:** Net maintenance requirements (NMR) of fattening yaks.

Items	Cu (mg/d)	Mn (mg/d)	Zn (mg/d)	Fe (mg/d)	Se (mg/d)	Co (mg/d)
Fattening yak	0.87	0.45	14.48	19.89	0.11	0.01

## 4 Discussion

### 4.1 Growth performance

Nutrient intake is fundamental for maintaining normal growth in animals (20). As feeding duration was extended and feeding level increased, FBW, SBW, and EBW all increased significantly, consistent with the results of Ji (21) and Cao (22). DMI in the AL group was also significantly higher than that in the M group, which may be attributed to the expansion of gastrointestinal tract volume and enhanced feeding capacity during growth, enabling individuals to consume more nutrients to support body weight gain (23). However, the difference in ADG between the M and AL groups did not reach statistical significance, possibly because both groups were fed ad libitum without a nutritional gradient, resulting in no significant difference in daily weight gain.

### 4.2 Copper distribution and requirements in fattening yaks

Copper plays key roles in hematopoiesis, cuproenzyme synthesis, hair melanogenesis, and skeletal development (24) and is primarily stored in the liver (18). Numerous studies have described Cu distribution in ruminants. Ji (21) reported that viscera (including blood) accounted for 84.24 and 69.78% of whole-body Cu in F1 Dorper × Small-tailed Han male and female lambs, respectively, with muscle being the next highest tissue. In crossbred Dorper rams, Ma (25) likewise found 83.16 to 88.90% of Cu in viscera (including blood), 3.06–7.09% in adipose, and 3.46–4.99% in muscle. In yak calves weighing 70–90 kg, Cao et al. (26) measured 66.80% of Cu in viscera (including blood) and 12.10% in muscle. In the present study of fattening yaks, copper contents in viscera (including blood) and muscle were 61.12 and 27.52%, respectively, with the muscle content being substantially higher than values reported in lambs and calves. This likely reflects differences in species and physiological stage.

For the NRG of copper, Cao (22) derived the equation for yak calves as NRG = 0.1154 × EBW^0.8350^, which gives a copper NRG range of 9.14–12.46 mg/kg EBW based on the EBW range of this study. Using the Nellore cattle model [NRG = 1.25 × EBW^0.33^ (27)] within this EBW range, the copper NRG was estimated to be 7.04–7.95 mg/kg EBW. The measured NRG for fattening yaks in this study (7.08–7.58 mg/kg EBW) was comparable to that of Nellore cattle but markedly lower than the calf-based estimate. This finding suggests that mineral requirement models developed for growing calves may overestimate the net copper requirement for growth of fattening yaks. This difference may be primarily associated with the more intensive growth metabolism of yak calves. During the rapid growth stage, yak calves exhibit active development of muscle and visceral tissues (28), accompanied by intensive metabolic activity. Because copper is involved in hematopoiesis, connective tissue synthesis, antioxidant enzyme function, and mitochondrial respiration (29), animals at this stage may require greater copper deposition and utilization. In contrast, fattening yaks gradually shift toward fat deposition as the expansion of metabolically active tissues slows down, reducing the net copper requirement per unit of body weight gain. In addition, yaks are raised under conditions of chronic high-altitude hypoxia on the Tibetan Plateau. Under hypoxic conditions, mitochondrial oxidative phosphorylation and antioxidant defense are closely associated with copper-dependent enzymes, including cytochrome c oxidase and superoxide dismutase (30, 31). High-altitude hypoxia can also increase oxidative stress and alter physiological adaptation in mammals (32, 33), potentially influencing copper metabolism and utilization in yaks.

For the NMR of copper, the ARC (19) value of 7.1 μg/kg BW·d^−1^ yields 1.63–2.27 mg/d for the present BW range, whereas the CSIRO (34) value of 4.0 μg/kg BW·d^−1^ corresponds to 0.92–1.28 mg/d. The measured Cu net maintenance requirement for fattening yaks was 0.87 mg/d in the present study, below the ARC-based estimate and closer to the CSIRO value.

### 4.3 Manganese distribution and requirements in fattening yaks

Manganese is an essential trace element present at low levels in the body, with concentrations in cattle and sheep carcasses ranging from 0.5 to 3.9 mg/kg dry matter (35). Its distribution varies by species and physiological stage. Ji (21) reported that in F1 Dorper × Small-tailed Han male and female lambs, Mn was mainly distributed in viscera (including blood, 63.67 and 52.65%), hair (10.57 and 22.63%), and muscle (10.18 and 15.01%). Ma (25) likewise indicated that in crossbred Dorper rams Mn was primarily in viscera (including blood, 67.92%), followed by muscle (11.63%), whereas Cao (22) found in yak calves that Mn was enriched mainly in bone (49.16%), hair (19.81%), and muscle (15.95%). In this study, manganese in fattening yaks was primarily distributed in bone (45.62%), viscera (including blood, 18.90%), and muscle (13.53%). The distribution pattern of manganese in yaks differed obviously from that in Dorper crossbred sheep, in which manganese was mainly deposited in viscera. In contrast, yaks predominantly accumulated manganese in bone, indicating a marked species difference. Compared with yak calves, the proportion of manganese in bone of fattening yaks decreased, while that in viscera increased, reflecting the effect of physiological stage on manganese distribution.

For the NRG of manganese, Zhang (36) derived the equation for the growth phase of yaks as NRG = 4.004 × EBW^−0.387^. When applied to the EBW range of this study, the estimated manganese NRG was 0.46–0.53 mg/kg EBW. In contrast, for fattening yaks in this study, the corresponding NRG was measured at 0.99–1.15 mg/kg EBW, which is higher than the value extrapolated from the growth phase yak model.

For the NMR of manganese, the theoretical value calculated using the NRC (37) recommended level for dairy cattle (2.00 μg/kg BW·d^−1^) corresponds to 0.46–0.64 mg/d for the body weight range of yaks in this study. The estimated NMR in this study was 0.45 mg/d, which is essentially consistent with the NRC (37) recommendation.

### 4.4 Zinc distribution and requirements in fattening yaks

Zinc plays key roles in protein and fatty acid metabolism and in free radical scavenging (38). Regarding tissue distribution, Cao (22) reported that in yak calves, zinc is primarily distributed in muscle (60.14%) and bone (25.67%) with viscera and blood accounting for only 8.52%. Similarly, Bo (39) found that in late-growth calves, muscle (54.89%), bone (15.87%), and viscera with blood (13.36%) are the main zinc storage sites. In the present study, fattening yaks showed zinc mainly distributed in muscle (49.78%), viscera with blood (24.57%), and bone (16.10%). Across these three developmental stages, the proportion of zinc in viscera and blood gradually increased (8.52% → 13.36% → 24.57%), muscle proportion remained relatively stable (~55–60%), and bone proportion fluctuated. This shift likely reflects a physiological transition from skeletal growth to metabolic and immune function maintenance, with zinc redistributing from bone to viscera to support enzyme systems and immune demands (40). Thus, muscle, viscera, and bone are the main zinc depots, with viscera zinc enrichment increasing with age.

For the NRG of zinc, ARC (19) recommends a range of 16 to 31 mg/kg BW for beef cattle. The zinc NRG formula derived by Cao (22) for yak calves is NRG = 98.0840 × EBW^−0.2416^. Based on the EBW range of yaks in this study, the calculated zinc NRG is 25.31–27.68 mg/kg EBW. In this study, the zinc NRG for fattening yaks was found to be 30.31 to 31.57 mg/kg EBW, which is higher than the value extrapolated from the growth-phase yak model and approaches or slightly exceeds the upper limit of the ARC (19) recommendation.

For the NMR of zinc, NRC (35) estimated endogenous urinary Zn loss in beef cattle at 12 μg/kg BW·d^−1^ (range 4–19 μg/kg BW·d^−1^). Estimates for dairy cattle maintenance were 53 μg/kg BW·d^−1^ (41), 55 μg/kg BW·d^−1^ (19, 37), and 45 μg/kg BW·d^−1^ (34). For the body weights in this study, these correspond to 2.76–3.84 mg/d, 12.19–16.96 mg/d, 12.65–17.60 mg/d, and 10.35–14.40 mg/d, respectively. The measured NMR for fattening yaks in the present study was 14.48 mg/d. This value is higher than beef cattle estimates that consider only urinary loss, but closer to those reported for lactating dairy cows.

### 4.5 Iron distribution and requirements in fattening yaks

Iron is a key trace element for the formation of hemoglobin and red blood cells, and its supplementation can increase milk yield, improve milk quality in dairy cows, and promote weight gain in beef cattle (42). Approximately 60 to 70% of body iron occurs as heme iron in the blood (18). Regarding tissue distribution, Ji (21) reported that in F1 Dorper × Small-tailed Han lambs, Fe was mainly in viscera including blood (66.99% in males; 56.15% in females), followed by bone (12.80%; 14.82%). In yak calves, Cao et al. (26) measured 54.80% of Fe in viscera including blood. In the present study, Fe in fattening yaks was primarily distributed in viscera including blood (63.42%) and bone (19.23%), consistent with these reports.

For the NRG of iron, NASEM (43) proposed 115 mg/kg EBW for beef cattle. Zhang (36) derived the equation for growing yaks as NRG = 117.318 × EBW^−0.042^, which gives 92.70–94.16 mg/kg EBW for the EBW range in this study. In fattening yaks of this study, the corresponding Fe requirement was 101.21–122.08 mg/kg EBW, which is close to the above values.

For the NMR of iron, studies are limited. Cao (22) reported 11.35 mg/d for yak calves. In this study, the estimated NMR for fattening yaks was 19.89 mg/d, higher than in calves. This difference likely reflects the greater iron stores required for oxygen transport in larger fattening yaks.

### 4.6 Selenium distribution and requirements in fattening yaks

Selenium participates in lipid redox processes and protects against oxidative damage (4). Studies on its tissue distribution in ruminants are limited. In yak calves, Se (22) was reported to be mainly in muscle (47.43%) and viscera including blood (23.85%). In fattening yaks of this study, Se was likewise concentrated in muscle (52.85%) and viscera (including blood, 29.65%).

For the NRG of selenium, Zhang (36) derived the equation for growing yaks as NRG = 0.058 × EBW^0.286^, which gives 0.26–0.29 mg/kg EBW for the EBW range in this study. For Nellore cattle (27), the model NRG = 1.07 × EBW^−0.07^ yields 0.72–0.74 mg/kg EBW over the present EBW range. In fattening yaks of this study, Se NRG was 0.23–0.31 mg/kg EBW, comparable to that of growing yaks but markedly lower than Nellore cattle. This difference may be related to the long-term adaptation of yaks to the selenium-deficient environment of the Tibetan Plateau (7). Such adaptation may be associated with changes in selenium metabolism and utilization, helping maintain antioxidant and metabolic functions under low-selenium conditions (7). Moreover, during the late fattening stage, tissue growth intensity decreases and fat deposition becomes more prominent, which may reduce selenium deposition requirements per unit of EBW gain.

For the NMR of selenium, Cao (22) reported 0.16 mg/d for yak calves. In this study, the estimated Se NMR for fattening yaks was 0.11 mg/d, slightly lower than the calf value.

### 4.7 Cobalt distribution and requirements in fattening yaks

Cobalt is essential for the synthesis of vitamin B_12_ (cobalamin), participates in hematopoiesis and immune modulation, and promotes growth and digestion in ruminants (5). Regarding tissue distribution, Cao (22) reported that in yak calves Co was mainly in bone (38.54%) with appreciable amounts in muscle (25.67%), viscera including blood (14.93%), and skin (12.42%). In fattening yaks of this study, Co was primarily distributed in muscle (27.57%) and bone (23.35%). This difference likely reflects age-related changes in body composition, as body weight and muscle mass increase, total cobalt in muscle surpassed that in bone.

For the NRG of cobalt, growing ruminants have higher vitamin B_12_ needs (18) and therefore greater Co requirements. Zhang (36) derived the equation for growing yaks as NRG = 0.004 × EBW^0.324^, which gives approximately 0.02 mg/kg EBW for the EBW range in this study. In the present study, the NRG for fattening yaks was 0.01 mg/kg EBW, similar to the value for growing yaks.

For the NMR of cobalt, studies are limited. Because the efficiency of deriving vitamin B_12_ from dietary Co is low, 84–98% of dietary Co appears in feces within 5–14 days (44). Cao (22) reported an NMR of 0.01 mg/d for yak calves. In the present study, the fattening yak value was the same (0.01 mg/d).

## 5 Conclusion

This study established EBW-based prediction models for estimating the net growth and maintenance requirements of trace elements in late-fattening yaks. Compared with values reported for yak calves, a greater proportion of whole-body Mn was distributed in bone tissue, whereas greater proportions of whole-body Zn and Co were distributed in visceral tissues and muscle, respectively, in late-fattening yaks. Net growth requirements (mg/kg EBW) were 7.08–7.58 for Cu, 0.99–1.15 for Mn, 30.31–31.57 for Zn, 101.21–122.08 for Fe, 0.23–0.31 for Se, and 0.01 for Co. Corresponding net maintenance requirements (mg/d) were 0.87, 0.45, 14.48, 19.89, 0.11, and 0.01, respectively. These EBW-based equations and requirement estimates provide reference values for precision trace-element supplementation in yak production. However, the models developed in this study are applicable only within the body-weight range of 230–320 kg; extrapolation beyond this range may reduce prediction accuracy and should therefore be undertaken with caution. Further studies involving wider body-weight ranges and diverse production conditions are needed to validate and refine these models, thereby improving their generalizability and reliability.

## Data Availability

The original contributions presented in the study are included in the article/Supplementary material, further inquiries can be directed to the corresponding authors.

## References

[1] Tariq M. Hameed A. Yak (*Bos grunniens*): the mammal of socio-economic importance in Gilgit-Baltistan, Pakistan Proceedings of the XXV International Grassland Congress (2023) Covington, Lexington International Grassland Congress 1406–1414

[2] Zhang Y Wang F. Industry survey and analysis report on China's yak market. Agricultural Products Market (2021) (23):54–55. Available online at: http://journal.crnews.net/ncpsczk/2021n/d23q/dcyj/945311_20220303060946.html (Accessed August 23, 2025).

[3] Cao Y Li L Zhang H. Study of blood clinical pathological changes of selenium deficiency of dairy cattle in intensive dairy farm. J Heilongjiang Bayi Agric Univ. (2016) 28:49–54. doi: 10.3969/j.issn.1002-2090.2016.06.010

[4] Huo B Wu T Song C-J Shen X-Y. Effects of selenium deficiency in alpine meadow on blood biochemical indexes and antioxidant systems of yaks. Chin J Anim Husb Vet Med. (2019) 46:1053–62. doi: 10.16431/j.cnki.1671-7236.2019.04.012

[5] Zhang R Cheng Z Zang C Cui C Zhang C Jiao Y . Supplementation of 5,6-dimethylbenzimidazole and cobalt in high-concentrate diet improves the ruminal vitamin B12 synthesis and fermentation of sheep. Fermentation (Basel). (2023) 9:956. doi: 10.3390/fermentation9110956

[6] Kennedy DG Young PB McCaughey WJ Kennedy S Blanchflower WJ. Rumen succinate production may ameliorate the effects of cobalt-vitamin B-12 deficiency on methylmalonyl CoA mutase in sheep. J Nutr. (1991) 121:1236–42. doi: 10.1093/jn/121.8.1236, 1677683

[7] Zhao K Huo B Shen X. Studies on antioxidant capacity in selenium-deprived Choko yaks in the Shouqu prairie. Biol Trace Elem Res. (2020) 199:3297–302. doi: 10.1007/s12011-020-02461-9, 33123866

[8] Duan J. *Research on major macromineral requirements and deposition-distribution patterns in yak calves* (master's thesis). Qinghai University, Xining (2023)

[9] Zhu X. Analysis of selenium content in yaks in the lake surrounding area of Qinghai Province. Chin Qinghai J Anim Vet Sci. (2005) 35:60. doi: 10.3969/j.issn.1003-7950.2005.04.040

[10] Cao J Zhao T-Z Zhang F Chen Y-W Fu X-L Deng C-L . Research on mineral nutrient requirements of yaks. Mod Anim Husb Sci Technol. (2024) 3:122–5. doi: 10.19369/j.cnki.2095-9737.2024.03.034

[11] Lofgreen G Garrett W. A system for expressing net energy requirements and feed values for growing and finishing beef cattle. J Anim Sci. (1968) 27:793–806. doi: 10.2527/jas1968.273793x

[12] Rodrigues JPP Lima JCM Castro MMD Valadares Filho S d C Chizzotti ML Campos MM . Macromineral requirements of Holstein calves. Pesq Agrop Brasileira. (2018) 53:522–5. doi: 10.1590/s0100-204x2018000400015

[13] MOA Breeding Standard for Beef Cattle (Standard No. NY/T 815-2004). Beijing: China Agriculture Press (2004). Available online at: https://std.samr.gov.cn/hb/search/stdHBDetailed?id=B89AE1B85644E2A8E05397BE0A0AF7F9 (Accessed August 23, 2025).

[14] Gotsick EE Smith SR Stanton VL Teutsch CD Henning JC. A comparison of four methods for determining pasture botanical composition. Grassl Res. (2025) 4:260–8. doi: 10.1002/glr2.70014

[15] AOAC. Official Methods of Analysis of AOAC International. 22nd ed. Oxford: Oxford University Press (2023).

[16] Van Soest PJ Robertson JB Lewis BA. Methods for dietary fiber, neutral detergent fiber, and nonstarch polysaccharides in relation to animal nutrition. J Dairy Sci. (1991) 74:3583–97. doi: 10.3168/jds.s0022-0302(91)78551-2, 1660498

[17] Galvani DB Pires CC Kozloski GV Wommer TP. Energy requirements of texel crossbred lambs. J Anim Sci. (2008) 86:3480–90. doi: 10.2527/jas.2008-1097, 18708598

[18] NRC. Nutrient Requirements of Small Ruminants: Sheep, Goats, Cervids, and New World Camelids. Washington, DC: National Academies Press (2007).

[19] ARC. The Nutrient Requirements of Ruminant Livestock. Farnham Royal: Commonwealth Agricultural Bureaux (1980).

[20] Peng T Wang Y Wang G Chen Y Yang Y. Effects of dietary energy and protein levels on growth performance, serum biochemical indices, slaughter performance and meat quality of Shanbei white cashmere goats. Chin J Anim Nutr. (2018) 30:2194–201. doi: 10.3969/j.issn.1006-267x.2018.06.022

[21] Ji S. *Study on the distribution patterns and requirement parameters of major minerals in Dorper × thin-tailed Han crossbred lambs (F1) aged 20–35 kg* (master's thesis). Chinese Academy of Agricultural Sciences, Beijing (2013)

[22] Cao J. *Nutrient requirements and deposit distribution of major trace minerals in yak calves* (master's thesis). Qinghai University, Xining (2023)

[23] Sun D-M Yin Y-Y Wu J-L Liu L-X Mao S-Y Liu J-H. Effect of early mashed and pelleted concentrate starter supplementation on animal performance and development of the gastrointestinal tract in lambs. Acta Pratacult Sin. (2020) 29:184–92. doi: 10.11686/cyxb2019432

[24] Yang S Hu P Chen D. Physiological function and growth-promoting mechanism of trace element copper. Feed Ind. (2004) 25:23–6. doi: 10.3969/j.issn.1001-991x.2004.07.001

[25] Ma X. *Energy, protein and main mineral requirements for the maintenance and growth of 35–50 kg Dorper × Jinzhong Crossbred ram lamb* (master's thesis). Shanxi Agricultural University, Taigu (2016).

[26] Cao J Zhang B Duan J Fu Y Li S Yang D . Study on mineral elements copper, iron and zinc nutrient requirement of 70 to 90 kg yak calves. Chin J Anim Nutr. (2023) 35:1705–15. doi: 10.12418/cjan2023.161

[27] Costa e Silva LF de Campos Valadares Filho S Engle TE Rotta PP Marcondes MI Silva FAS . Macrominerals and trace element requirements for beef cattle. PLoS One. (2015) 10:e0144464. doi: 10.1371/journal.pone.0144464, 26657049 PMC4681427

[28] Yohe T Dennis T Buss L Croft E Quigley J Hill T . Performance and visceral tissue growth and development of Holstein calves fed differing milk replacer allowances and starch concentrations in pelleted starter. J Dairy Sci. (2022) 105:4099–115. doi: 10.3168/jds.2021-21286, 35221069

[29] López-Alonso M Miranda M. Copper supplementation, a challenge in cattle. Animals. (2020) 10:1890. doi: 10.3390/ani10101890, 33076570 PMC7602799

[30] Eikens W Kienitz C Jones P Thone C. Copper trafficking to the mitochondrion and assembly of copper metalloenzymes. Biochim Biophys Acta, Mol Cell Res. (2006) 1763:759–72. doi: 10.1016/j.bbamcr.2006.03.002, 16631971

[31] Horn D Barrientos A. Mitochondrial copper metabolism and delivery to cytochrome c oxidase. IUBMB Life. (2008) 60:421–9. doi: 10.1002/iub.50, 18459161 PMC2864105

[32] Dosek A Ohno H Acs Z Taylor AW Radak Z. High altitude and oxidative stress. Respir Physiol Neurobiol. (2007) 158:128–31. doi: 10.1016/j.resp.2007.03.013, 17482529

[33] Storz JF Scott GR Cheviron ZA. Phenotypic plasticity and genetic adaptation to high-altitude hypoxia in vertebrates. J Exp Biol. (2010) 213:4125–36. doi: 10.1242/jeb.048181, 21112992 PMC2992463

[34] CSIRO. Nutrient Requirements of Domesticated Ruminants. Melbourne: CSIRO Publishing (2007).

[35] NRC. Nutrient Requirements of Beef Cattle. 7th ed. Washington, DC: National Academies Press (2000).

[36] Zhang L. *Deposition of trace mineral elements in yaks during the growing period and nutrient requirement studies* (master's thesis). Qinghai University, Xining (2023)

[37] NRC. Nutrient Requirements of Dairy Cattle. 5th ed. Washington, DC: National Academy Press (1975).

[38] Tang Z Chen Y Duan D Li H Li Z Ren T . Physiological function of micromineral zinc and its application in pig breeding. China Anim Husb Vet Med. (2022) 49:4228–38. doi: 10.16431/j.cnki.1671-7236.2022.11.013

[39] Bo Z. *Study on main minerals distribution and requirement parameters of yak calves at late growth stage* (master's thesis). Qinghai University, Xining (2022).

[40] Wessels I Fischer HJ Rink L. Dietary and physiological effects of zinc on the immune system. Annu Rev Nutr. (2021) 41:133–75. doi: 10.1146/annurev-nutr-122019-120635, 34255547

[41] Weigand E Kirchgessner M. Factorial estimation of the zinc requirement of lactating dairy cows. Z Tierphysiol Tierernahr Futtermittelkd. (1982) 47:1–9. doi: 10.1111/j.1439-0396.1982.tb01228.x, 7048796

[42] Harris DC. Anaemia in housed lambs. Vet Rec. (1995) 136:500. doi: 10.1136/vr.136.19.500-a, 7645191

[43] NASEM. Nutrient Requirements of Beef Cattle. 8th ed. Washington, DC: National Academies Press (2016).

[44] Looney J Gille G Preston R Graham E Pfander W. Effects of plant species and cobalt intake upon cobalt utilization and ration digestibility by sheep. J Anim Sci. (1976) 42:693–8. doi: 10.2527/jas1976.423693x, 1262282

