# Supplementary material for: Tissue distribution and net maintenance and growth requirements of key trace minerals in fattening yaks (Bos grunniens) on the Qinghai–Tibetan plateau
Source: Front Vet Sci. 2026 Jul 3;13:1864547. doi: 10.3389/fvets.2026.1864547 (PMC13377711; doi:10.3389/fvets.2026.1864547)
Supplement: Supplementary file 1 [file Data_Sheet_1.pdf]

Supplementary Material

Supplementary Table S1. Main operating parameters for inductively coupled plasma optical emission spectrometry (ICP-OES)

Rinse pump rate (rpm)	Analysis pump rate (rpm)	Pump stabilization time (s)	RF power (W)	Auxiliary gas flow rate (L/min)	Nebulizer gas flow rate (L/min)	Vertical observation height (mm)
55	55	5	1150	0.5	0.55	12.0

Supplementary Table S2. Main operating parameters for inductively coupled plasma mass spectrometry (ICP-MS)

Plasma power (W)	Sampling depth (mm)	Interface pressure (hPa)	Spray chamber temperatu re (℃)	Cooling gas flow rate (L/min)	Carrier gas flow rate (L/min)	Collision /Reaction gas flow rate (r/min)	RF frequenc y (MHz)
1548.6	5	1.51	2.1	14.04	10.85	40	1.938

Supplementary Table S3. Linear calibration equations and coefficients of determination for mineral elements

Items	Calibration equation (Y = a·X + b)[1)]	Coefficient of determination (R^2)
Cu（μg/L）	Y=9378.4444 X - 366.6491	0.9994
Mn（μg/L）	Y=2705.6046 X + 78.4083	0.9997
Zn（μg/L）	Y=1760.5301 X + 1806.3378	0.9902
Fe（μg/L）	Y=130.2657 X + 8.3112	0.9990
Se（μg/L）	Y=40.8945 X + 8.1807	0.9983
Co（μg/L）	Y=10785.5389 X + 364.5102	0.9994

[1)]Y denotes instrument response; X denotes concentration in μg/L; a and b are regression coefficients.

Supplementary Table S4. Tissue Growth of Adult Yaks at Different Slaughter Stages and Feeding Levels

Items	Groups[1)]					SEM	P-value
	BL	M	AL	IR70	IR40		
Total bone weight (kg)	28.04^d	31.69^c	37.80^a	36.57^a	34.45^b	0.76	<0.001
Total muscle weight (kg)	95.27^d	108.46^c	134.50^a	132.70^a	124.87^b	3.24	<0.001
Total fat weight (kg)	17.42^d	21.04^c	27.60^a	25.00^b	22.38^c	0.73	<0.001
Total viscera weight including blood (kg)	28.45^c	37.17^b	43.58^a	41.44^a	40.44^a	1.13	<0.001
Total hide weight (kg)	13.25^b	14.79^b	18.49^a	17.48^a	17.02^a	0.42	<0.001
Total hair weight (kg)	3.22^b	3.71^b	4.60^a	4.44^a	4.39^a	0.20	<0.001

[1)]Different lowercase letters above the same row indicate significant differences (P < 0.05), whereas identical letters or no letter indicate no significant difference (P > 0.05).

BL: baseline slaughter group; M: midterm slaughter group; AL: ad libitum terminal group; IR70: 70% of ad libitum intake group; IR40: 40% of ad libitum intake group.

Supplementary Table S5. Dry matter content of tissues in fattening yaks

Items	Groups[1)]					SEM	*P*-value
	BL	M	AL	IR70	IR40		
Bone	72.81	73.18	72.29	74.54	73.54	0.55	0.790
Muscle	31.14	32.49	33.91	31.39	31.11	0.47	0.266
Fat	81.01	81.54	82.32	81.51	81.02	0.17	0.084
Blood & viscera	30.85	30.30	31.37	30.51	29.86	0.51	0.793
Hide	38.24	39.82	37.54	38.66	37.65	0.42	0.458
Hair	—	—	—	—	—	—	—

[1)]Different lowercase letters above the same row indicate significant differences ($P < 0.05$), whereas identical letters or no letter indicate no significant difference ($P > 0.05$).

BL: baseline slaughter group; M: midterm slaughter group; AL: ad libitum terminal group; IR70: 70% of ad libitum intake group; IR40: 40% of ad libitum intake group.

Supplementary Table S6. Tissue distribution of copper (Cu, mg) in fattening yaks

Groups[1)]	Tissues						Total
	Bone	Muscle	Fat	Blood & viscera	Hide	Hair	
BL	15.33^{c}	296.94^{d}	87.21	720.23^{d}	14.87^{d}	18.33^{c}	1152.96^{c}
Proportion (%)	1.33	25.75	7.57	62.47	1.29	1.59	—
M	23.19^{b}	377.63^{cd}	94.45	863.99^{cd}	22.89^{c}	23.90^{c}	1405.77^{b}
Proportion (%)	1.65	26.87	6.72	61.42	1.63	1.70	—
AL	28.37^{a}	532.91^{a}	99.33	1025.97^{a}	31.69^{a}	33.95^{a}	1751.02^{a}
Proportion (%)	1.62	30.40	5.67	58.56	1.81	1.94	—
IR70	24.70^{ab}	457.15^{ab}	96.81	995.53^{ab}	29.75^{ab}	31.41^{ab}	1635.28^{a}
Proportion (%)	1.51	27.96	5.92	60.87	1.82	1.92	—
IR40	20.75^{b}	389.61^{bc}	92.24	908.96^{bc}	25.58^{b}	25.14^{bc}	1461.92^{b}
Proportion (%)	1.42	26.65	6.31	62.17	1.75	1.72	—
Mean proportion (%)	1.50	27.52	6.43	61.12	1.66	1.77	—
SEM	1.41	19.86	5.03	30.00	2.01	1.95	46.97

[1)]Different lowercase letters within the same column indicate significant differences ($P < 0.05$), whereas identical letters or no letters indicate no significant difference ($P > 0.05$).

BL: baseline slaughter group; M: midterm slaughter group; AL: ad libitum terminal group; IR70: 70% of ad libitum intake group; IR40: 40% of ad libitum intake group. Total (mg): sum of element contents across all tissues; Proportion (%): proportion of each tissue's element content relative to the whole-body total; Mean proportion (%): average proportion of each tissue's element content relative to the whole-body total.

Supplementary Table S7. Tissue distribution of manganese (Mn, mg) in fattening yaks

Groups[1)]	Tissues						Total
	Bone	Muscle	Fat	Blood & viscera	Hide	Hair	
BL	69.35^{c}	16.62^{c}	5.31	21.33^{c}	16.16^{c}	7.38^{c}	136.15^{d}
Proportion (%)	50.93	12.21	3.90	15.66	11.87	5.42	—
M	77.89^{b}	23.05^{bc}	6.00	31.83^{b}	20.99^{b}	12.29^{b}	172.05^{c}
Proportion (%)	45.27	13.40	3.49	18.50	12.20	7.14	—
AL	97.27^{a}	32.29^{a}	7.68	44.28^{a}	26.34^{a}	15.49^{a}	223.37^{a}
Proportion (%)	43.55	14.46	3.44	19.82	11.79	6.94	—
IR70	87.71^{ab}	28.58^{ab}	7.50	40.19^{a}	24.36^{ab}	12.77^{ab}	201.12^{b}
Proportion (%)	43.61	14.21	3.73	19.98	12.11	6.35	—
IR40	86.25^{b}	25.82^{ab}	6.85	39.51^{ab}	22.57^{ab}	11.90^{b}	192.91^{bc}
Proportion (%)	44.71	13.38	3.55	20.48	11.70	6.17	—
Mean proportion (%)	45.62	13.53	3.62	18.90	11.93	6.40	—
SEM	2.98	1.61	0.42	2.16	0.99	0.70	6.40

[1)]Different lowercase letters within the same column indicate significant differences (P < 0.05), whereas identical letters or no letters indicate no significant difference (P > 0.05).

BL: baseline slaughter group; M: midterm slaughter group; AL: ad libitum terminal group; IR70: 70% of ad libitum intake group; IR40: 40% of ad libitum intake group. Total (mg): sum of element contents across all tissues; Proportion (%): proportion of each tissue's element content relative to the whole-body total; Mean proportion (%): average proportion of each tissue's element content relative to the whole-body total.

Supplementary Table S8. Tissue distribution of zinc (Zn, g) in fattening yaks

Groups$^{1)}$	Tissues						Total
	Bone	Muscle	Fat	Blood & viscera	Hide	Hair	
BL	1.13^{d}	4.24^{b}	0.19^{b}	1.79^{c}	0.14^{b}	0.20^{d}	7.69^{c}
Proportion (%)	14.86	55.54	2.45	23.48	1.8	2.57	—
M	1.43^{cd}	4.29^{b}	0.30^{b}	2.11^{b}	0.20^{ab}	0.31^{cd}	8.65^{b}
Proportion (%)	16.53	49.59	3.47	24.39	2.31	3.58	—
AL	1.84^{a}	5.38^{a}	0.49^{a}	2.73^{a}	0.25^{a}	0.52^{a}	11.21^{a}
Proportion (%)	16.41	48.00	4.37	24.35	2.23	4.64	—
IR70	1.73^{ab}	5.02^{a}	0.46^{a}	2.65^{a}	0.22^{a}	0.46^{ab}	10.55^{a}
Proportion (%)	16.40	47.60	4.36	25.12	2.09	4.36	—
IR40	1.61^{bc}	4.76^{ab}	0.40^{ab}	2.52^{ab}	0.21^{ab}	0.38^{bc}	9.88^{a}
Proportion (%)	16.30	48.18	4.05	25.51	2.12	3.85	—
Mean proportion (%)	16.10	49.78	3.74	24.57	2.11	3.80	—
SEM	0.05	0.15	0.05	0.13	0.01	0.03	0.32

$^{1)}$Different lowercase letters within the same column indicate significant differences ($P < 0.05$), whereas identical letters or no letters indicate no significant difference ($P > 0.05$).

BL: baseline slaughter group; M: midterm slaughter group; AL: ad libitum terminal group; IR70: 70% of ad libitum intake group; IR40: 40% of ad libitum intake group.

Total (g): sum of element contents across all tissues; Proportion (%): proportion of each tissue's element content relative to the whole-body total; Mean proportion (%): average proportion of each tissue's element content relative to the whole-body total.

Supplementary Table S9. Tissue distribution of iron (Fe, g) in fattening yaks

Groups[1)]	Tissues						Total
	Bone	Muscle	Fat	Blood & viscera	Hide	Hair	
BL	2.62^{d}	1.00^{d}	0.26^{b}	8.21^{c}	0.39^{d}	0.61^{d}	13.08^{d}
Proportion (%)	20.03	7.65	1.99	62.77	2.98	4.66	—
M	3.35^{c}	1.14^{cd}	0.39^{a}	10.52^{b}	0.55^{cd}	0.81^{c}	16.76^{c}
Proportion (%)	19.99	6.80	2.33	62.77	3.28	4.83	—
AL	4.20^{a}	1.45^{a}	0.47^{a}	13.92^{a}	0.88^{a}	1.20^{a}	22.12^{a}
Proportion (%)	18.99	6.56	2.12	62.93	3.99	5.42	—
IR70	3.87^{ab}	1.30^{ab}	0.46^{a}	13.24^{a}	0.78^{ab}	1.02^{ab}	20.67^{ab}
Proportion (%)	18.72	6.29	2.23	64.05	3.77	4.93	—
IR40	3.53^{bc}	1.26^{bc}	0.42^{a}	12.38^{b}	0.67^{bc}	0.91^{bc}	19.17^{b}
Proportion (%)	18.41	6.57	2.19	64.58	3.50	4.75	—
Mean proportion (%)	19.23	6.77	2.17	63.42	3.50	4.91	—
SEM	0.14	0.04	0.02	0.32	0.05	0.05	0.73

[1)]Different lowercase letters within the same column indicate significant differences ($P < 0.05$), whereas identical letters or no letters indicate no significant difference ($P > 0.05$).

BL: baseline slaughter group; M: midterm slaughter group; AL: ad libitum terminal group; IR70: 70% of ad libitum intake group; IR40: 40% of ad libitum intake group.

Total (g): sum of element contents across all tissues; Proportion (%): proportion of each tissue's element content relative to the whole-body total; Mean proportion (%): average proportion of each tissue's element content relative to the whole-body total.

Supplementary Table S10. Tissue distribution of selenium (Se, mg) in fattening yaks

Groups[1)]	Tissues						Total
	Bone	Muscle	Fat	Blood & viscera	Hide	Hair	
BL	1.60^{b}	15.93^{b}	0.91^{c}	6.44^{b}	0.77^{b}	0.56	26.23^{d}
Proportion (%)	6.10	60.73	3.45	24.55	2.94	2.13	—
M	3.56^{a}	18.67^{ab}	1.15^{bc}	12.19^{a}	1.65^{a}	0.58	37.81^{c}
Proportion (%)	9.42	49.38	3.04	32.24	4.36	1.53	—
AL	4.14^{a}	25.70^{a}	1.86^{a}	14.01^{a}	1.80^{a}	0.67	48.20^{a}
Proportion (%)	8.59	53.32	3.86	29.07	3.73	1.39	—
IR70	4.05^{a}	22.48^{a}	1.68^{a}	13.68^{a}	1.71^{a}	0.65	44.24^{ab}
Proportion (%)	9.15	50.81	3.80	30.92	3.87	1.47	—
IR40	3.93^{a}	20.81^{a}	1.54^{ab}	13.08^{a}	1.63^{a}	0.63	41.62^{bc}
Proportion (%)	9.44	50.00	3.70	31.43	3.92	1.51	—
Mean proportion (%)	8.55	52.85	3.57	29.65	3.76	1.62	—
SEM	0.33	2.00	0.19	0.77	0.15	0.04	2.37

[1)]Different lowercase letters within the same column indicate significant differences ($P < 0.05$), whereas identical letters or no letters indicate no significant difference ($P > 0.05$).

BL: baseline slaughter group; M: midterm slaughter group; AL: ad libitum terminal group; IR70: 70% of ad libitum intake group; IR40: 40% of ad libitum intake group.

Total (mg): sum of element contents across all tissues; Proportion (%): proportion of each tissue's element content relative to the whole-body total; Mean proportion (%): average proportion of each tissue's element content relative to the whole-body total.

Supplementary Table S11. Tissue distribution of cobalt (Co, mg) in fattening yaks

Groups[1)]	Tissues						Total
	Bone	Muscle	Fat	Blood & viscera	Hide	Hair	
BL	0.55[c]	0.66[c]	0.42[c]	0.38[c]	0.10[c]	0.06[c]	2.17[c]
Proportion (%)	25.38	30.39	19.17	17.66	4.83	2.67	—
M	0.60[bc]	0.72[bc]	0.51[b]	0.50[b]	0.26[b]	0.08[b]	2.68[b]
Proportion (%)	22.52	26.92	19.01	18.63	9.82	3.06	—
AL	0.74[a]	0.89[a]	0.58[a]	0.59[a]	0.40[a]	0.12[a]	3.31[a]
Proportion (%)	22.22	26.71	17.63	17.72	12.10	3.59	—
IR70	0.72[a]	0.85[a]	0.55[ab]	0.58[a]	0.36[a]	0.11[a]	3.18[a]
Proportion (%)	22.77	26.66	17.43	18.18	11.40	3.52	—
IR40	0.70[a]	0.80[a]	0.51[b]	0.53[ab]	0.30[ab]	0.11[a]	2.95[ab]
Proportion (%)	23.79	27.17	17.33	18.07	10.02	3.62	—
Mean proportion (%)	23.35	27.57	18.11	18.03	9.64	3.30	—
SEM	0.03	0.04	0.02	0.02	0.03	0.01	0.10

[1)]Different lowercase letters within the same column indicate significant differences ($P < 0.05$), whereas identical letters or no letters indicate no significant difference ($P > 0.05$).

BL: baseline slaughter group; M: midterm slaughter group; AL: ad libitum terminal group; IR70: 70% of ad libitum intake group; IR40: 40% of ad libitum intake group.

Total (mg): sum of element contents across all tissues; Proportion (%): proportion of each tissue's element content relative to the whole-body total; Mean proportion (%): average proportion of each tissue's element content relative to the whole-body total.

Supplementary Table S12. Daily mineral deposition (MR) in yaks (mg/d)

Items	Groups[1)]				SEM	*P*-value
	M	AL	IR70	IR40		
Cu	5.41[a]	5.29[a]	3.79[b]	1.94[c]	0.34	＜0.001
Mn	1.12[a]	1.30[a]	0.85[b]	0.31[c]	0.09	＜0.001
Zn	45.50[a]	44.70[a]	34.05[b]	10.54[c]	3.28	＜0.001
Fe	89.36[b]	100.80[a]	76.91[c]	55.00[d]	3.99	＜0.001
Se	0.44[a]	0.44[a]	0.24[b]	0.16[c]	0.03	＜0.001
Co	0.014[a]	0.015[a]	0.009[b]	0.003[c]	0.001	＜0.001

[1)]Different lowercase letters above the same row indicate significant differences ($P < 0.05$), whereas identical letters or no letter indicate no significant difference ($P > 0.05$).

M: midterm slaughter group; AL: ad libitum terminal group; IR70: 70% of ad libitum intake group; IR40: 40% of ad libitum intake group.
